# Supplementary material for: Modeling glioblastoma heterogeneity as a dynamic network of cell states
Source: Mol Syst Biol. 2021 Sep 16;17(9):e10105. doi: 10.15252/msb.202010105 (PMC8444284; doi:10.15252/msb.202010105)
Supplement: Supplementary file 6 — Source Data for Figure 5 [file MSB-17-e10105-s004.zip › Figure5A_sourcedata/GSEA_3071/hallmarks_stateII.GseaPreranked.1623742536692/HALLMARK_ALLOGRAFT_REJECTION.html]

Details for gene set HALLMARK\_ALLOGRAFT\_REJECTION[GSEA]

|  || Dataset | state23071 |
| Phenotype | NoPhenotypeAvailable |
| Upregulated in class | na\_pos |
| GeneSet | HALLMARK\_ALLOGRAFT\_REJECTION |
| Enrichment Score (ES) | 0.40228137 |
| Normalized Enrichment Score (NES) | 1.830761 |
| Nominal p-value | 0.0023640662 |
| FDR q-value | 0.011430124 |
| FWER p-Value | 0.079 |
Table: GSEA Results Summary

  

Fig 1: Enrichment plot: HALLMARK\_ALLOGRAFT\_REJECTION      
 Profile of the Running ES Score & Positions of GeneSet Members on the Rank Ordered List

  

| PROBE | GENE SYMBOL | GENE\_TITLE | RANK IN GENE LIST | RANK METRIC SCORE | RUNNING ES | CORE ENRICHMENT || 1 | IL11 |  |  | 5 | 1.740 | 0.0888 | Yes |
| 2 | BRCA1 |  |  | 49 | 1.249 | 0.1428 | Yes |
| 3 | LIF |  |  | 64 | 1.184 | 0.2006 | Yes |
| 4 | INHBA |  |  | 196 | 0.876 | 0.2137 | Yes |
| 5 | EGFR |  |  | 198 | 0.874 | 0.2587 | Yes |
| 6 | CCND3 |  |  | 390 | 0.683 | 0.2471 | Yes |
| 7 | IL18 |  |  | 427 | 0.666 | 0.2726 | Yes |
| 8 | ITGB2 |  |  | 476 | 0.639 | 0.2939 | Yes |
| 9 | FLNA |  |  | 483 | 0.637 | 0.3253 | Yes |
| 10 | NPM1 |  |  | 544 | 0.605 | 0.3419 | Yes |
| 11 | SOCS1 |  |  | 593 | 0.581 | 0.3601 | Yes |
| 12 | DEGS1 |  |  | 688 | 0.536 | 0.3647 | Yes |
| 13 | ABCE1 |  |  | 716 | 0.527 | 0.3853 | Yes |
| 14 | TRAF2 |  |  | 830 | 0.489 | 0.3829 | Yes |
| 15 | ELF4 |  |  | 910 | 0.468 | 0.3877 | Yes |
| 16 | GZMB |  |  | 948 | 0.459 | 0.4023 | Yes |
| 17 | EIF3J |  |  | 1159 | 0.405 | 0.3716 | No |
| 18 | EIF3A |  |  | 1196 | 0.397 | 0.3833 | No |
| 19 | TAP2 |  |  | 1215 | 0.394 | 0.3992 | No |
| 20 | NME1 |  |  | 1343 | 0.368 | 0.3870 | No |
| 21 | EIF3D |  |  | 1405 | 0.357 | 0.3905 | No |
| 22 | EIF5A |  |  | 1461 | 0.346 | 0.3948 | No |
| 23 | GLMN |  |  | 1634 | 0.319 | 0.3690 | No |
| 24 | HIF1A |  |  | 1667 | 0.315 | 0.3774 | No |
| 25 | ETS1 |  |  | 1721 | 0.308 | 0.3803 | No |
| 26 | RIPK2 |  |  | 1892 | 0.286 | 0.3533 | No |
| 27 | CAPG |  |  | 2029 | 0.269 | 0.3337 | No |
| 28 | GALNT1 |  |  | 2349 | -0.264 | 0.2689 | No |
| 29 | RPS9 |  |  | 2353 | -0.265 | 0.2818 | No |
| 30 | ABI1 |  |  | 2598 | -0.301 | 0.2374 | No |
| 31 | UBE2D1 |  |  | 3063 | -0.389 | 0.1433 | No |
| 32 | NCK1 |  |  | 3156 | -0.415 | 0.1421 | No |
| 33 | HDAC9 |  |  | 3288 | -0.453 | 0.1333 | No |
| 34 | ST8SIA4 |  |  | 3406 | -0.500 | 0.1304 | No |
| 35 | TGFB2 |  |  | 3774 | -0.766 | 0.0797 | No |
Table: GSEA details [plain text format]

  

Fig 2: HALLMARK\_ALLOGRAFT\_REJECTION: Random ES distribution      
 Gene set null distribution of ES for **HALLMARK\_ALLOGRAFT\_REJECTION**
